# Metastatic basal cell carcinoma: Case series and literature review^☆^^☆☆^^★^

**DOI:** 10.1016/j.bjorl.2025.101619

**Published:** 2025-05-28

**Authors:** Mariana Gonçalves Rodrigues, Aline Vieira de Lucena, Gabriela Alves Domingues, Caroline Marques de Aquino, Deborah Yukiko Otto, André Bandiera de Oliveira Santos, Luiz Paulo Kowalski

**Affiliations:** a Universidade de São Paulo (USP), Faculdade de Medicina, Disciplina de Cirurgia de Cabeça e Pescoço, São Paulo, SP, Brazil; b Universidade Nove de Julho (UNINOVE), Faculdade de Medicina, São Paulo, SP, Brazil; c Universidade de São Paulo (USP), Faculdade de Medicina, Departamento de Radiologia, São Paulo, SP, Brazil

**Keywords:** Carcinoma, Basal cell, Neoplasm metastasis, Head and neck neoplasms

## Abstract

**Objective:**

To present a metastatic head and neck basal cell carcinoma (BCC) series treated by a university teaching service between 1993 and 2021.

**Methods:**

A review of medical records of 500 patients with high-risk BCC in the head and neck was performed. Tumors with a diameter greater than or equal to 6 mm in the “H” area of the face or greater than 10 mm in other head and neck areas; recurrences; sclerodermiform, micronodular and metatypical subtypes; and perineural invasion were considered factors indicating high-risk.

**Results:**

Seven cases of metastatic head and neck BCC were diagnosed. The mean age at diagnosis of the primary tumor was 49.8 years, and the median time between the diagnosis of the primary tumor and that of metastases was 3 years (ranging from 1 to 20 years). The main site of metastasis was the lymph nodes. The main modalities of metastases treatment were surgery and radiotherapy.

**Conclusion:**

BCC metastasis is a rare manifestation of a frequent disease. Post-treatment follow-up should include routine clinical examination of the neck for all patients and imaging for the diagnosis of metastases in symptomatic patients.

**Level of evidence:**

Level IV.

## Highlights

• The basal cell carcinoma metastases are a disease's late manifestation.
• The main site of metastasis was the lymph nodes.
• The main modalities of metastases treatment were surgery and radiotherapy.
• The basal cell carcinoma metastasis is a rare manifestation of a frequent disease.
• Clinical follow-up should include neck physical examination for all patients and imaging only if suspicion of distant metastasis.

## Introduction

Basal Cell Carcinoma (BCC) is the most common malignancy in humans.^1^ In Europe, the incidence of BCC ranges from 44.4 to 128 cases per 100,000 inhabitants, while the overall incidence is estimated at 10 million cases per year,^2^ mainly in white individuals. Its first clinical description dates back to 1827, when the Irish surgeon Arthur Jacob reported cases of “rodent ulcers” on the faces of three individuals.^3^ The main risk factors for BCC are exposure to ultraviolet radiation and genetic factors. Mutations in the hedgehog signaling pathway account for 90% of BCC cases.^2^ These tumors have a slow clinical evolution with local invasion and rarely result in the development of metastases.^2^

In a review of the literature published in 2020, Bisceglia et al.^2^ identified 915 reports of metastatic BCC. Lattes and Kessler^4^ proposed the following criteria for the diagnosis of metastatic BCC: (1) The primary lesion should originate from the skin and not in mucous membranes or salivary glands; (2) Metastases should be distant from the primary lesion and not be of the same extent; and (3) The primary lesion and metastasis should be similar in terms of histological type. These criteria can help distinguish metastatic BCC from other possible primary tumors or locally advanced BCC.

About 54% of metastatic BCC originates from tumors in the head and neck region, and the main site of metastasis is the lymph nodes, followed by the lungs and bones.^5^ Metastatic BCC demonstrates very aggressive behavior. Although the time interval between the onset of the disease and the occurrence of metastasis is approximately 8 years, once the presence of a metastatic growth is diagnosed, life expectancy is up to 24 months.^5^ Several risk factors have been identified for the development of metastases, including size, depth, and site of the primary tumor; recurrent tumors; perineural invasion; intravascular invasion; and histological subtype.^6^, ^7^, ^8^ Other factors, such as previous radiotherapy, increased expression of smooth muscle actin, and decreased expression of E-cadherin, may also contribute to the metastatic potential of BCCs.^9^

In the present study, we present a series of seven cases of metastatic BCC treated at the Head and Neck Surgery department of a referral service between 1993 and 2021.

## Methods

Medical records of patients diagnosed with high-risk BCC were retrieved. All cases treated between 1993 and March 2021 were reviewed. High-risk tumors were considered those with a diameter greater than or equal to 6 mm in the “H” area of the face or those greater than 10 mm in other areas of the head and neck; recurrences; sclerodermiform, micronodular and metatypical subtypes; and with perineural invasion. Five hundred patients were identified as having high-risk BCC. In this series, the Lattes and Kessler criteria were used for the diagnosis of metastatic BCC.^4^ A literature review was also performed using the PubMed database. The terms “metastatic basal cell carcinoma” and “head and neck” were used in the search, and the filters “Review”, “Systematic Review” and “Meta-Analysis” were applied. Only papers published in English were researched. Single case reports were excluded.

The present study was approved by the ethics and research committee of the Institution (743366).

## Results

Seven patients were diagnosed with metastatic BCC during the study period. Four patients were female (57,1%). The mean age at diagnosis of the primary tumor was 49.8 years (ranging from 30 to 66 years-old), and the median time between diagnosis of the primary tumor and metastasis was 3 years (ranging from 1 to 20 years). One patient (case 6) had Gorlin-Goltz syndrome (dysplastic basal cell nevus syndrome), which is considered a risk factor for skin tumors. In all cases, metastatic disease was presented in the context of a local recurrence instead of as a primary manifestation of the disease. The main site of metastasis was the lymph nodes (six cases), followed by bones (one case). There was also a cerebellar metastasis and one pulmonary metastasis but both without histological evidence. The main modality of treatment of metastases was surgery and adjuvant radiotherapy (five cases). Two patients’ cause of death was directly related to metastatic BCC (Table 1). The most relevant results of the literature review are presented in Table 2.

**Table 1:** Patients diagnosed with metastatic BCC according to gender, age at diagnosis of the primary tumor, site of the primary lesion, site of metastasis, time interval between the primary tumor and metastasis, metastasis treatment modality, survival time after the diagnosis of metastasis and cause of death.

Case	Gender F/M	Age (years)	Primary tumor site	Local recurrence	Time to metastasis (years)	Metastasis site	Metastasis treatment	Survival time after metastasis	Cause of death
1	M	30	Pre-auricular	Yes	6	Lymph node	Surgery and RDT	7 years	Lung Cancer
2	M	46	Ear	Yes	3	Lymph node	Surgery and RDT	16 years	COVID-19
3	M	50	Nose	Yes	1	Lymph node	Surgery and RDT	10 years	Unknown
4	F	56	Malar	Yes	3	Lymph node	Surgery	1 month	Post-operative complications
5	F	47	Nose	Yes	20	Lymph node	Surgery and RDT	>4 years	Does not apply
6	F	54	Face	Yes	6	Bone	Chemotherapy	3 months	BCC metastasis
7	F	66	Pre-auricular	Yes	3	Lymph node	Surgery and RDT	1 year	Does not apply

M, Male; F, Female.

**Table 2:** Literature review results according to year of publication, study design, number of cases, site of BCC metastasis, time interval from primary site diagnostic and metastasis, and survival time after metastasis.

Author	Famer and Helwig^10^	von Domanus and Stevens^11^	Snow et al.^6^	Tavin et al.^12^	McCusker et al.^5^	Tang et al.^13^	Biseeglis et al.^2^
Publication	1980	1984	1994	1995	2014	2017	2020
Periodic	Cancer	Journal of the American Academy of Dermatology	Cancer	Laryngoscope	European Journal of Cancer	Australas J Dermatol	Advances in Anatomic Pathology
Study design	Case series	Case series and review	Case series and review	Case series	Review	Case series and review	Review
Number of cases	17 (13 head and neck)	5 (case series, 80% head and neck)/ 170 (review 70% head and neck)	5 (case series/ 65 review)	6	100 (56% head and neck)	8 (63% head and neck)	692 (67 %–85 % head and neck)
Site of metastasis	Lung, bone, lymph nodes, liver spleen	Lymph nodes, lungs, and bones	Regional lymph nodes, skin and submandibular gland	Subcutaneous tissue, cervical lymph nodes, bone, and lung	Lymph nodes, bone lung salivary gland, liver, skin or soft tissue	Lymph nodes, bone, lug and skin	Lymph nodes, salivary glands and liver
Time interval until metastasis	0‒30 years/ 9.6 years (mean)/ 6.0 years (median)	9 years (median)	12 years	1.5–14 years	8 years (mean) 6 (median)	0‒16 years (median 2 years)	1‒25 years (median 9−5 years)
Survival time after metastasis	1.6 years (mean)	8 months (median)	0.3–7 years	‒	54 months, 87 months (nodal metastasis) and 24 months (distant metastasis)	1 death 4.5 years	87 months (nodal metastasis and 24 months (distant metastasis)

## Case 1

Male, 30 years old, diagnosed with BCC in the right preauricular region. Submitted to resection at an external medical service (information on pathological examination not available). He presented a local recurrence the following year that was treated with new local resection and radiotherapy (dose and number of sessions are unknown; the treatment was performed by an external service). His BCC evolved with a new local recurrence 6 years later, for which he underwent surgical resection. Pathological examination revealed that it was an ulcerated BCC of 1.2 cm in the largest axis with impaired (deep) resection margins. While the patient waiting for the procedure for margin enlargement, the surgeon identified a cervical lymph node, for which Fine Needle Aspiration Puncture (FNAB) was positive for neoplastic cells, consistent with BCC. Then, the patient underwent a new surgery: preauricular margin enlargement, parotidectomy, zygomatic arch resection, temporal muscle resection, neck dissection, and regional flap rotation. The pathological examination showed BCC with a solid pattern; sclerodermiform and metatypical, infiltrating the skin in an area close to the previous surgery; parotid, fibro adipose tissue, and skeletal muscle, with perineural invasion; infiltration of the zygomatic bone; resection margins free of neoplasia; and the presence of metastasis in 32 of the 57 dissected lymph nodes (13 at level II, one at level III, 11 at level IV, and seven at level V), with extra capsular extension. He received adjuvant therapy (6,000 cGy). The patient attained good control of the skin disease and died 7 years after the diagnosis of metastasis due to a non-small cell lung neoplasm.

## Case 2

**Fig. 1:**
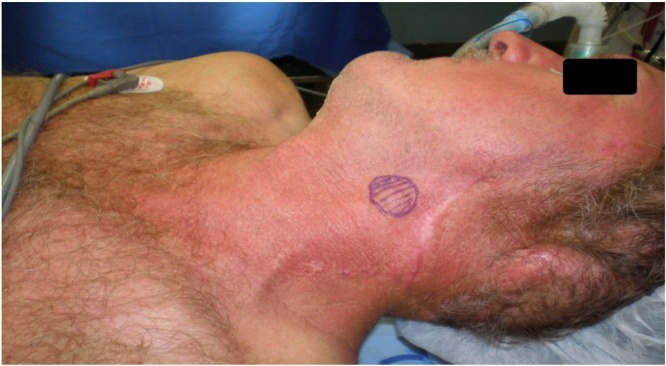
Patient positioned for left neck dissection due to lymph node metastasis at level II. The palpable lymph node was marked with a hatched circle.

**Fig. 2:**
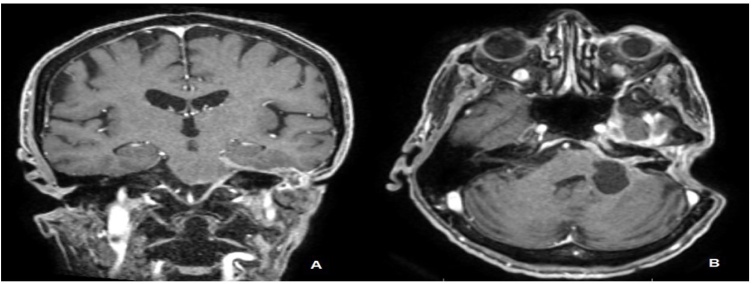
Magnetic resonance imaging of a T1-weighted skull. Paquimeningeous thickening along the cistern of the left cerebellar point angle, as well as the interior of the internal auditory canal on the left side, is pictured. It is associated with an expansive lesion, apparently cystic, in continuity with the tissue observed inside the left internal auditory canal, measuring approximately 3.1 × 1.9 cm in the largest axes in the axial plane. (A) Coronal cut. (B) Axial cut.

Male, 46 years-old, rural worker, underwent a biopsy of a skin lesion of 2.5 cm in the left ear, which revealed sclerodermiform BCC. Six months later, a new skin lesion biopsy in the left temporal region confirmed the diagnosis of solid BCC. He was then submitted to resection of the left auricular pavilion and biopsy of the suspected cervical lymph node, for which pathological examination by frozen section did not show metastatic disease. Three years later, he presented with local recurrence and was treated with resection of the recurrent lesion and left superficial parotidectomy. During postoperative follow-up, FNAB of a level II cervical lymph node was performed, showing metastasis of BCC (Fig. 1). The metastatic disease was treated surgically through a neck dissection and adjuvant radiation therapy (information about the dose and number of sessions are not available). The pathological examination revealed the involvement of three out of 12 dissected lymph nodes. Eighteen years after the diagnosis of the primary lesion, the patient experienced weakness in the lower limbs and imbalance. He also presented with difficulty to speak and eat, requiring help for all instrumental and basic activities of life; however, his cognition was preserved. A magnetic resonance imaging of the skull (Fig. 2A and 2B) showed a neoplastic lesion in the cerebellum (plausible metastasis of a skin tumor, not confirmed by histopathological examination), for which treatment was performed with dexamethasone and palliative radiotherapy, but with no functional improvement. The patient died 1 year later of severe acute respiratory syndrome caused by SARS-CoV-2.

## Case 3

**Fig. 3:**
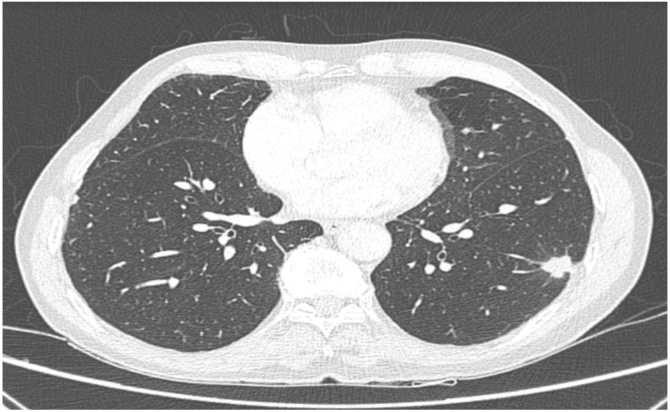
Computed tomography of the chest. There are bilateral spiculated pulmonary nodules, with the largest occurring in the superior segment of the left lower lobe, at 1.5 cm.

Male, 50 years old, diagnosed with metatypical BCC after curettage of a skin lesion in the nose. The following year, he was diagnosed with new skin lesions in the left posterior cervical region, upper lip, and nasal dorsum, as well as a suspected cervical lymph node. He was submitted to resection of the skin lesions and a neck dissection. Pathological report confirmed sclerodermiform and adenoid BCC measuring at 6.5 cm, with free margins and no perineural invasion in the posterior cervical lesion; 2.2 cm sclerodermiform BCC with free margins in an upper lip lesion; and 1 cm sclerodermiform and solid BCC with free margins in the nasal lesion. Metastasis was found in three of 10 dissected lymph nodes, with extra capsular extension. The patient underwent adjuvant therapy at a dose of 6,000 cGy. After 2 years, he presented with a local recurrence of BCC in the nasal region, which was treated with local resection. Six years after the diagnosis of the first skin lesion, four pulmonary nodules with spiculated contours were diagnosed bilaterally (Fig. 3); they were considered BCC pulmonary metastases, but without histological proof. The lung lesions were treated with chemotherapy (paclitaxel and carboplatin, eight cycles). The following year, he presented with a recurrence of BCC in a retro auricular lymph node, with ulceration in the mastoid region. The surgical salvage was contraindicated due to the involvement of the internal carotid. He received systemic treatment with carboplatin and paclitaxel regimen (D1, D8, and D15 every 4-weeks), which was later combined with weekly methotrexate. The patient died 11 years after the diagnosis of the primary lesion at an external health service. The cause of death was not reported.

## Case 4

Female, 56 years old, diagnosed with 2.5 cm sclerodermiform BCC in the left malar region, without perineural or angiolymphatic invasion by excisional biopsy. She demonstrated local recurrences in the next 3 years, all of which were treated with surgical resection. The last recurrence was locally advanced and was treated with craniofacial resection and neck dissection. Pathological examination showed a nodular, adenoid and sclerodermiform BCC, measuring 2.7 cm, with the presence of perineural invasion, focally compromised margins, and involvement of two of the 29 lymph nodes analyzed, without extra capsular extension. The patient did not receive adjuvant treatment, as she died one month after surgery due to postoperative clinical complications (septic shock due to pneumonia associated with mechanical ventilation).

## Case 5

Female, with a history of an ulcerated facial lesion resection when she was 47 years old. Twenty years later, she presented with an ulcer-infiltrative lesion of approximately 3.5 × 4.0 cm in the right malar region and nasal wing, a nodular lesion in the nasal dorsum of 1 cm in diameter, and another ulcerated lesion in the upper lip of 1 cm next to the area of the previous resection. Scars of a left Indian flap rotated to the nasal dorsum and the presence of an enlarged left submandibular lymph node of 2.0 cm were also found. She was diagnosed with solid BCC after a biopsy of the nasal lesion. Given the evidence of recurrence, she underwent a total rhinectomy, a selective neck dissection (levels I, II, and III), and microsurgical reconstruction. The pathological report revealed a lesion of 3.5 × 3.0 cm, with a maximum thickness of 0.7 cm, a basaloid component with an extensive epidermoid component, microscopic extension to adipose and muscle tissue, ulceration and perineural invasion, and free margins. Lymph node metastasis demonstrated an IB level of 1.7 × 1.0 × 0.8 cm. The patient underwent adjuvant radiotherapy with a total dose of 5,000 cGy in the surgical bed and left cervical drainage bed. The patient lacked signs of recurrence of oncologic disease until the fourth year of outpatient medical follow-up.

## Case 6

A female patient with a history of Gorlin-Goltz syndrome (dysplastic basal cell nevus syndrome) had her first BCC diagnosed on the right hemiface when she was 25 years old. At 60 years of age, she was submitted to right- orbital exenteration due to BCC in the face with 6 years of evolution, which invaded the nasal septum and right eyeball. The pathological report showed a sclerodermiform BCC of 8 cm with compromised margins. She received adjuvant treatment (unknown modality). She later experienced local recurrence, with involvement of the left orbit and bone metastasis in the right femur, which was confirmed by biopsy and treated with chemotherapy with Taxol (eight cycles, without therapeutic response). The patient died three months after the diagnosis of metastasis while in palliative care due to clinical complications of the metastatic disease.

## Case 7

Female, 66 years-old, with a nodule in the left preauricular region resected at an external service (solid BCC with free surgical margins). Six months after resection, she developed a new nodule in the left neck, which was also resected (pathological report: subcutaneous cellular tissue infiltrated by undifferentiated carcinoma). Three years after the last resection, she presented with a tumor growth on the left cervical scar and peripheral left facial paralysis. The patient underwent tumor resection in the left parotid region, total parotidectomy, mastoidectomy, and neck dissection of levels I–V. Pathological report revealed recurrent adenoid and sclerodermiform BCC with a solid infiltrative pattern, perineural invasion, and surgical margins free of neoplasia. Metastasis was present in one of the 23 lymph nodes analyzed. Adjuvant treatment with radiotherapy (dose: 4,800 cGy) was performed in the left parotid region. The last consultation recorded occurred 1 year after the diagnosis of lymph node metastasis, when there was no clinical suspicion of disease recurrence. The patient did not follow up after that point.

## Discussion

Metastatic BCC is a rare occurrence. McCusker et al.^5^ conducted a review of the cases reported between 1981 and 2011, identifying 50 patients with BCC metastases. von Domarus and Stevens^11^ conducted a similar review for a previous period, with 170 cases studied. The mean ages at diagnosis of the primary tumor in the above-mentioned studies were 54.5 years and 45 years, respectively, whereas the mean age in the present report was 49.8 years. Contrary to what had been described in the literature reviews, in which men were predominant, the proportion between female and male patients was similar in this series of cases. The median time between the diagnosis of the primary tumor and the finding of BCC metastasis in the previous studies was 6 and 9 years, respectively, whereas in the current study, the median was3 years (ranging from 1 to 20 years). The main site of metastasis described in the literature was the lymph nodes, which was confirmed in the present study.

Pulmonary and bone metastasis are the most frequently described sites of BCC metastasis besides the lymph nodes. In a study by McCusker et al.,^5^ the distribution of patients treated with surgery (40.4%), chemotherapy (36.2%), and radiotherapy (42.6%) for distance metastases for BCC were more uniform, whereas in the present study, there was a predominance of surgical treatment with adjuvant radiotherapy.

In this series of cases, two patients (28.5%) died within three months of the diagnosis of metastasis: in one of them (case 6), the metastasis was in the bones, and in the other (case 4), death occurred due to postoperative complications of extensive surgery. Among the five patients who had survived at least one year after the diagnosis of metastasis, two died before reaching five years of survival after metastasis treatment. Two patients died of causes unrelated to metastatic BCC: non-small cell lung cancer and COVID-19. One patient’s cause of death was not reported in medical records, as he died in another service; however, given that his BCC was locally advanced, with lymph node involvement and with probable pulmonary metastasis, it is possible that his death was related to skin cancer. In the literature, the mean survival time after the diagnosis of distance disease ranges from 8 to 24 months.^5^, ^11^

Bisceglia et al.^2^ published a literature review on metastatic BCC in 2020, which considered the studies mentioned above in addition to other cases and series of cases reports of more current cases, reaching 915 reported cases. The literature review confirmed previous findings, such as the higher frequency in males and the main location of the primary tumor being the head and neck (67%–85%). The occurrence of metastatic BCC in cases where the primary tumor was less than 1 cm was considered anecdotal. That study also reiterated the lymph nodes, especially cervical lymph nodes, as the main site of distant metastasis (> 50%), followed by pulmonary nodules (28%–33%) and bones (20%–24%).

In 2012, new treatment possibilities for metastatic or locally advanced BCC were approved. Vismodegib was the first target therapy approved by the Food and Drug Administration (FDA), and it operates in the hedgehog signaling pathway, which is abnormally activated in most BCCs.^8^ In this series of cases, no patient had access to target therapy. In most cases, the diagnosis of metastasis occurred before the approval of Vismodegib by the FDA; however, even nowadays, the Brazilian Public Health System (Sistema Único de Saúde ‒ SUS) still does not provide Vismodegib to its users, except for cases in research protocols.

It is necessary to mention limitations of the present study regarding data collection. During the period studied, some information was lost during the process of migration from physical records to electronic medical records. The Hospital where our study was conducted is a regional reference institution and receives patients referred from various health services in the country. When biopsy or initial treatment is not performed at this referencial center, some information from original medical records may not be completely transmitted. Despite these caveats, the authors reiterate that all patients reported in this study met the criteria for metastatic BCC.

## Conclusion

In conclusion, despite its rare occurrence, metastatic BCC is aggressive and should be considered in patients with high-risk BCC. It is essential to evaluate cervical lymph nodes by physical examination in all follow-up visits. Due to the rarity of distant metastases, staging tests are only recommended in symptomatic patients.

## Financial support

None.

## Declaration of competing interest

The authors declare no conflicts of interest.
